# The Gait Design and Trajectory Planning of a Gecko-Inspired Climbing Robot

**DOI:** 10.1155/2018/2648502

**Published:** 2018-04-22

**Authors:** Xuepeng Li, Wei Wang, Shilin Wu, Peihua Zhu, Fei Zhao, Linqing Wang

**Affiliations:** ^1^Robotics Institute, Beihang University, Beijing 100191, China; ^2^School of Mechanical Engineering and Automation, Beihang University, Beijing 100191, China; ^3^FAW-Volkswagen Automotive Co., Ltd., Changchun, Jilin 130000, China

## Abstract

Inspired by the dynamic gait adopted by gecko, we had put forward GPL (Gecko-inspired mechanism with a Pendular waist and Linear legs) model with one passive waist and four active linear legs. To further develop dynamic gait and reduce energy consumption of climbing robot based on the GPL model, the gait design and trajectory planning are addressed in this paper. According to kinematics and dynamics of GPL, the trot gait and continuity analysis are executed. The effects of structural parameters on the supporting forces are analyzed. Moreover, the trajectory of the waist is optimized based on system energy consumption. Finally, a bioinspired robot is developed and the prototype experiment results show that the larger body length ratio, a certain elasticity of the waist joint, and the optimized trajectory contribute to a decrease in the supporting forces and reduction in system energy consumption, especially negative forces on supporting feet. Further, the results in our experiments partly explain the reasonability of quadruped reptile's kinesiology during dynamic gait.

## 1. Introduction

Wall-climbing robots can move and work on a vertical wall to complete various tasks, which have attracted much attention of researchers around the world and have wide application fields including antiterrorism, postdisaster rescue, engineering test, and maintenance and inspection for hazardous environment [1–4]. Compared to the wheeled robots and caterpillar robots, the multilegged climbing robots have a distinct advantage of strong adaptability to unknown environment and uneven wall. Certainly, we must own the fact that they are limited by low velocity and large energy consumption. Here, it becomes very important to address gait design and trajectory planning of multilegged climbing robots, especially for bioinspired climbing robots.

Generally, gaits of multilegged climbing robot could be classified into two kinds by leg raise sequence and stability: the quasistatic gait and dynamic gait. As the name implies, the quasistatic gait shows that the whole robot system maintains static balance during the climbing process, which is easy to ensure the behavior of robot [5]. Many previous works have been carried out to promote development of climbing robots, such as the Climbing Mini-Whegs, the Waalbot II [6], the RiSE [7, 8], the Stintov, and the StickyBot [9, 10], characterized with pivot joints on the legs. These climbing robots can be applied in many challenging environments such as tree, glasses, cabinets, or concrete surfaces. However, their works are limited to relatively low velocities due to the quasistatic gait.

With the development of biomimetics, the dynamic gait which is closer to actual biologic locomotion has been applied to improve vertical climbing efficiency and reduce power consumption. To describe the dynamic gait, some models are abstracted from the biologic locomotion pattern, including the spring-mass (SM) model [11], the ubiquitous Spring-Loaded Inverted Pendulum (SLIP) model, the Lateral-Leg Spring (LLS) model [12], and the F-G model [13]. As expected, the climbing robots adopting dynamic gait performed an excellent performance from the perspective of high speed and low energy cost [14, 15]. However, there are still inevitable questions about the stability of climbing motion. With the DynoClimber and ROCR as an example, the lack of supporting feet makes both of them easily rotate around the vertical axis parallel with the climbing surface and leads to the weak stability during pendulum.

Trajectory planning for multilegged climbing robots refers to two major steps. Firstly, the trajectory solution for a required motion task could be obtained based on the forward and inverse kinematics. Furthermore, it is to ensure that the final path satisfies the needs of the desired conditions, such as minimum path, time, and energy consumption. Wang et al. investigated optimal attaching and detaching trajectory for wall climbing which guarantee reliability of the climbing robot [16]. Chen et al. operated the leg trajectories of the leg-wheel hybrid robot in a periodic manner on each step during stair climbing in view of a series of similar geometry constraints and limitation of the joint power density [17]. Akinfiev and Armada analyzed the influence of gravity on trajectory planning for climbing robots [18]. These works have improved the climbing performance of robots to a certain extent.

In our previous work, we have proposed the GPL (Gecko inspired mechanism with a Pendular waist and Linear legs) model and verified its feasibility [19]. The GPL model inspired by the F-G model consists of two rigid bodies (upper part and lower part with tail), four linear legs with spring buffers, and a passive waist joint. And it aims at explaining the relationships of the locomotion dynamics, the variables of the movement, and the parameters of the mechanism for sprawl quadruped climbing animals. To further develop dynamic gait and reduce energy consumption of climbing robots based on the GPL model, the gait design and trajectory planning will be addressed in this paper.

The organization of this paper is as follows: firstly, kinematics and dynamics of GPL are derived systematically and the conditions of gait reuse are obtained in the second section. And the singularity and feasible region of the waist are analyzed in detail. Then, the effects of structural parameters on the supporting forces are explored, including body length ratio, driving angle, and elasticity of the waist joint. Moreover, the trajectory of the waist is optimized based on system energy consumption. To testify our analysis, a bioinspired robot is developed and the prototype experiments are performed. Finally, conclusions are drawn and the future work is described.

## 2. Gait Design and Continuity Analysis

### 2.1. Kinematics and Dynamics of GPL

**Figure 1:**
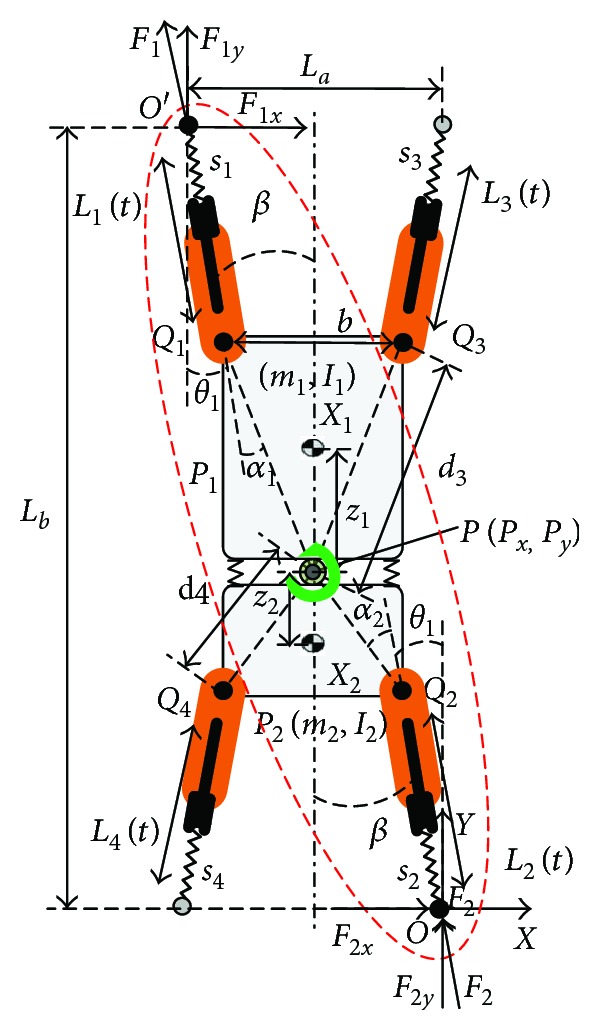
The GPL and its dynamic model derived from gecko. The red dotted line indicates the current adherent mechanism. The black dots on the foot mean the corresponding diagonal legs are attached with the climbing surface, while the gray dots mean the corresponding diagonal legs are swinging. Here, *L*_*a*_ and *L*_*b*_ are the horizontal and vertical distances between the diagonal stance feet, respectively. *L*_*i*_(*t*) and *x*_*P*_ denote the length of linear legs *L*_*i*_ and the position of *P*, which can be controlled. Let *m*_*j*_, *X*_*j*_, and *I*_*j*_(*j* = 1, 2) be mass, the position of the center, and the inertia moment tensor matrix of *P*_*j*_ in coordinate {*O*}, respectively. *Q*_*j*_, *d*_*j*_, *θ*_*i*_, and *α*_*j*_ denote the leg's fixed point on *P*_*j*_, the distance from *x*_*P*_ to *Q*_*j*_, the angle between leg and the vertical line, and the angle between corresponding leg and line *PQ*_*j*_, respectively. *Β* and *α*_*j*_ are constant values. *z*_*j*_ denotes the distance from *x*_*P*_ to the center of *P*_*j*_.

As illustrated in Figure 1, the GPL model abstracted from the gecko's morphology and kinesiology is composed of four linear legs *L*_*i*_ (*i* = 1–4), an upper part rigid body *P*_1_, a lower part rigid body *P*_2_, and a passive revolute joint called waist *P*. Here, the tail of the bionic prototype is very light and ignored to simplify the dynamic analysis. According to bionic research [25], the driving angle *β* between the driving force along the legs and the long axis of the body is almost constant during a moving cycle. Here, *β* keeps a constant value (10°). The front legs *L*_1_, *L*_3_ and rear legs *L*_2_, *L*_4_ are fixed on the upper and lower rigid body, respectively. Namely, the leg and the corresponding rigid body have the same angular velocity. In order to facilitate the analysis and design, waist *P* fixed on the upper part *P*_1_ is treated as a reference point of locomotion for the model. The lower body can revolve about the waist freely. To imitate gecko's wrist, one passive rotary joint is designed at the end of each leg, which allows the leg to rotate on the climbing surface. The GPL's locomotion can be realized by the legs' extending or contracting motion, cooperating with alternation of feet's attachment and wring. It notes that the waist has a certain elasticity by linking two rigid bodies with two linear springs.

**Figure 2:**
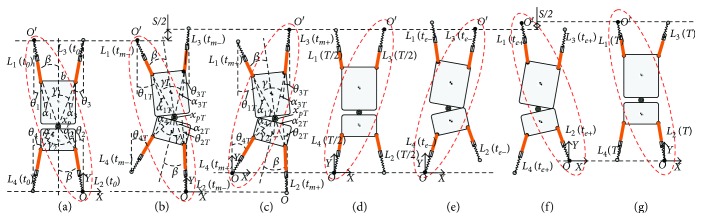
The trot gait inspired by gecko's climbing for the GPL model. Here, red dotted lines denote that the corresponding mechanisms are executing the active movement. (a)–(c) show the gait movement principle during half one gait. (a) The initial state of trot gait. *L*_1_ and *L*_2_ are adherent; *L*_3_ and *L*_4_ are swing. (b) *L*_1_ contracts and *L*_2_ extends; *L*_3_ and *L*_4_ are swing; and the waist swings as a pendulum and moves forward half stride *S*/2. (c) The role transformation of corresponding adherent and swinging diagonal feet. (d)–(e) The two processes are similar with (a) and (b). And the waist moves forward the other half stride *S*/2. (f) The role transformation status. (g) The initial state for the next gait.

The trot gait inspired by gecko's climbing is adopted for the GPL model, as shown in Figure 2. One stride of the trot gait during a stable climbing movement can be divided into two symmetrical phases. In one phase, the left front leg and the right rear leg begin to establish attachment with the climbing surface. Then, the left front leg contracts and the right rear leg extends, pulling the body upward, with other legs executing opposite motion. Meanwhile, the waist swings as a pendulum about the stance foot. Next, the other one executes the symmetrical motion.

In view of the symmetry of gait, we choose the procedure of one phase for analysis and apply the result to the next symmetrical phase, as shown by red dotted lines in Figure 1. Thus, the GPL model can be treated as a planar five-bar mechanism RPRPR with two degrees of freedom during one phase.

Let {*O*} and {*O*′} be a coordinate frame *O* − *XY* fixed on the right rear at *O* and left front foot at *O*′, respectively. The waist *P* rotates with the upper part around the stance foot *O*′. The pose of GPL can be expressed by by either **q**_*L*_ = [*L*_1_*L*_2_]^*T*^, **x**_*P*_ = [*P*_*x*_ *P*_*y*_]^*T*^, or **X***j* = [*Pxj* *Pyj*]^*T*^ as below (details can be found in [20]).

(1) $$\begin{array}{c} x_{p}=\left[\begin{array}{c} P_{x}\\ \frac{\left(\Phi \left(L_{2}\right)-\Phi \left(L_{1}\right)-2L_{a}P_{x}+C\right)}{2}L_{b} \end{array}\right],\\ {\dot{x}}_{p}=J{\dot{q}}_{L},\\ X_{j}=\left[\begin{array}{c} P_{x}\\ P_{y} \end{array}\right]+\left[\begin{array}{c} \sin\left(\theta_{j}-\beta \right)z_{j}\\ {\left(-1\right)}^{j+1}\cos\left(\theta_{j}-\beta \right)z_{j} \end{array}\right], \end{array}$$

where **J** is a 2 × 2 Jacobian matrix.

(2) $$\begin{array}{c} J=\frac{1}{L_{b}\times P_{x}+L_{a}\times P_{y}}\left[\begin{array}{ll} {mP}_{y}n & \left(L_{b}-P_{y}\right)\\ \begin{array}{cc} -{mP}_{x} & n\left(L_{a}+P_{x}\right) \end{array} \end{array}\right]. \end{array}$$

Based on (1) and (2), when given the length *q*_*L*_ of linear legs, the position *x*_*P*_ of the waist can be solved and vice versa, which lays the foundation for trajectory planning.

For further dynamic analysis of GPL, driving forces on the linear legs are analyzed, which significantly determine the control and performance of the robot. According to Autumn et al.'s research [21], energy loss during a rapid locomotion was generally attributed to the deceleration forces produced by the supporting feet. In that case, gravity and the gecko's legs decelerate the climbing within each step, resulting in velocity fluctuation. Therefore, it is worth analyzing the supporting forces on foot.

Let **F**_*j*_ be the active force applied on the linear legs. Let *E*_*K*_ and *E*_*P*_ be the kinetic energy and potential energy of the template; it can be calculated as follows:

(3) $$\begin{array}{c} E_{k}\left(q_{L},{\dot{q}}_{L}\right)=\frac{1}{2}\overset{j}{\underset{1}{\sum }}\left[I_{j}^{2}{\dot{\theta }}_{j}+m_{j}\left({\dot{P}}_{xj}+{\dot{P}}_{yj}\right)\right],\\ E_{p}\left(q_{L}\right)=E_{ps}+E_{pg}, \end{array}$$

where

(4) $$\begin{array}{c} E_{ps}=\frac{1}{2}\left(k_{w}\sigma_{w}^{2}+\overset{j}{\underset{1}{\sum }}k_{j}{\left(L_{j}-L_{sj}\right)}^{2}\right),\\ E_{pg}=\overset{j}{\underset{1}{\sum }}m_{j}{gP}_{y}. \end{array}$$

Here, the *E*_*ps*_ and *E*_*pg*_ denote the elastic potential energy and the gravitational potential energy of the template, respectively. *k*_*j*_ is the stiffness coefficient of the axil legs *L*_*j*_. *k*_*w*_ and *σ*_*w*_ are the stiffness coefficient and torsional angle of the pendular waist, respectively.

According to Lagrange dynamical equation, driving force **τ** = [*F*_1_*F*_2_]^*T*^, generated by the stretchout and drawback of the legs, can be calculated as follows:

(5) $$\begin{array}{c} \tau =\frac{d}{dt}\left(\frac{\partial T}{\partial {\dot{q}}_{L}}\right)-\frac{\partial T}{\partial q_{L}},\\ T=E_{k}\left(q_{L},{\dot{q}}_{L}\right)+E_{p}\left(q_{L}\right). \end{array}$$

Therefore, the joint power of the robot and system energy consumption in actual application of the climbing robot can be obtained as follows:

(6) $$\begin{array}{c} P_{j}\left(t\right)=\frac{R_{a}}{k_{M}^{2}\eta^{2}N_{G}^{2}}F_{j}^{2}\left(t\right)+\frac{{\dot{L}}_{j}\left(t\right)F_{j}\left(t\right)}{\eta },\\ E\left(t\right)=\overset{n}{\underset{j=1}{\sum }}\int_{t_{0}}^{t_{m}}P_{j}\left(t\right)dt\;\left(j=1,2\right). \end{array}$$

### 2.2. Condition of Gait Reuse

Generally, the gait designed for the robot has obvious periodicity in view of motion stability and control complexity. When a symmetrical gait is adopted, the trajectory planning for a step could be reused in the entire regular motion trajectory, especially linear trajectory or circle trajectory. Unlike the other quadruped robots, there is only a controllable local degree of freedom on the foot for the GPL model. This means that when two diagonal legs are attached with the climbing surface, the remaining legs are in an approximately free state, leading to the uncontrollable poses of the corresponding feet. In other words, the floating feet poses cannot reach the necessary conditions needed for the trot gait reuse when the GPL model is in the transitional state from one phase to the other one in one stride (*S*). In this paper, the conditions of GPL gait reuse are analyzed and the gait continuity is ensured by taking linear trajectory as an example.

Figures 2(a)–(c) describe the gait movement principle during half one gait. When *t* = *t*_0_, the climbing robot is in the initial state, in which the diagonal legs with the black dot on the foot are attached with the climbing surface and the diagonal legs with the blank dot on the foot are swinging; see Figure 2(a). Namely, the left front leg and the right rear leg begin to establish attachment with the climbing surface. Then, the left leg contracts and the right leg extends, pulling the body upward. When *t* = *t*_*m*—_, the climbing robot is in the transitional state; see Figure 2(b). It is obvious that the position of waist *P* moves from *x*_*P*_ to *x*_*PT*_, and the distance in vertical direction is just half a stride. When *t* = *t*_*m*+_, the other diagonal mechanism indicated by red dotted lines starts to execute the active movement, as shown in Figure 2(c). Now, the current state becomes the initial state of the next half one gait. In the same way, the right front leg and the left rear leg begin to establish attachment with the climbing surface and execute a similar movement. So, the state transition is realized from the former half one gait to the next one. Of course, the following conditions need to be satisfied.

Set the initial time *t* = *t*_0_ and the terminal time *t* = *t*_*m*_ during half a gait. Let *x*_*P*_ and *x*_*PT*_, *L*_*i*_(*t*0) and *L*_*i*_(*tm*), and *θ*_*i*_ and *θ*_*iT*_ (*i* = 1–4) be the position of the waist, length of linear legs, and angle between leg and the corresponding vertical line in the initial and transitional states of gait, respectively. Let *γj* (*j* = 1, 2) be the angle between line *PQ*_*j*_ and *PQ*_*j*+2_, keeping a constant value. Let *S* be the vertical stride of one gait.

To realize the gait continuity, the following constraints need to be satisfied when the following phase repeats the trajectory of the previous phase in one stride.

(a) Structure parameter constraints
It is mainly considered that the prototype design should keep bilateral symmetry. Thus,
  (7) $$\begin{array}{c} \alpha_{j}=\alpha_{j+2},\\ d_{j}=d_{j+2}\;\left(j=1,2\right). \end{array}$$
(b) Leg pose constraints
These constraints include two aspects: feet angle and leg length. Here, the leg lengths can be actively controlled by motors.
  (8) $$\begin{array}{c} \theta_{j}=\theta_{\left(j+2\right)T},\\ L_{j}\left(t_{0}\right)=L_{\left(j+2\right)}\left(t_{m}\right)\;\left(j=1,2\right). \end{array}$$
(c) Waist position constraints about central symmetry
This is determined by the trot gait adopted by the GPL model. Thus,
  (9) $$\begin{array}{c} x_{p}+x_{pT}=-\frac{L_{a}}{2}. \end{array}$$
Based on the geometric constraints, there exists a fixed relationship as follows:
  (10) $$\begin{array}{c} \theta_{j}+\alpha_{j}+\theta_{j+2}+\alpha_{j+2}=\gamma_{j},\\ \theta_{jT}+\alpha_{j}+\theta_{\left(j+2\right)T}+\alpha_{\left(j+2\right)T}=\gamma_{j}\;\left(j=1,2\right). \end{array}$$
According to (1), (7), (8), (9), and (10), the conditions of gait reuse and continuity for the GPL model can be obtained as follows.
  (11) $$\begin{array}{c} x_{p}=f_{1}\left(\theta_{1},\theta_{2}\right),\\ x_{pT}=f_{1}\left(\theta_{1T},\theta_{2T}\right),\\ f_{1}\left(\theta_{1T},\theta_{2T}\right)+f_{1}\left(\theta_{1},\theta_{2}\right)=-\frac{L_{a}}{2}, \end{array}$$
  where
  (12) $$\begin{array}{c} \theta_{jT}=2\beta_{j}-\theta_{j}. \end{array}$$
From the above, it can be seen that the position of the waist has specific limitations to realize the gait continuity during the climbing process, rather than any position.

## 3. Trajectory Planning of GPL

### 3.1. Singularity and Feasible Region of the Waist

For the trajectory planning, singularity and workspace are both inevitable problems. Let ∣**J**∣ denote the determinant of the Jacobian matrix **J** of the GPL model. When ∣**J**∣ → ∞ is satisfied, the singularity of the GPL model occurs.

(13) $$\begin{array}{c} \;|\;J\;|\;\to \infty \Leftrightarrow L_{b}\times P_{x}+L_{a}\times P_{y}=0. \end{array}$$

Obviously, the singularity of the robot will occur when the position of the waist is located on the diagonal anomaly line from (13). In this case, the driving forces on each supporting foot will be infinite theoretically. In order to address this issue, the waist trajectory should avoid intersection with the diagonal anomaly line. According to (5) and (6), it suggests that the farther the waist trail keeps away from the diagonal line linking the supporting feet, the smaller the maximum force on the axial legs. This rule should be considered during trajectory planning of the waist for the robot.

As we know, trajectory planning must be restricted to the corresponding workspace. Here, the waist *x*_*P*_ is selected as the reference point for trajectory planning. The feasible region of the waist can be obtained according to previous analysis. Let **W** ∈ **R**^2^ denote the feasible region of the waist. Let *P*_*x*min_ and *P*_*x*max_ be the minimum and maximal values of *P*_*x*_ for a certain *P*_*y*_ within the feasible region of the waist, respectively.

From (1), the relation between *P*_*x*_ and *P*_*y*_ can be derived as follows:

(14) $$\begin{array}{c} P_{x}=-d_{2}\sin\alpha_{2}\sec\theta_{2}-P_{y}\tan\theta_{2}. \end{array}$$

Given a large range [*P*_*y*min_, *P*_*y*max_] of *P*_*y*_ beyond the feasible region, *P*_*x*min_ and *P*_*x*max_ can be confirmed by the below constraints.

(15) $$\begin{array}{c} \mathrm{Minimize}\to \left\{\begin{array}{l} P_{x\min}=\min\left\{P_{x}\right\},\\ P_{x\max}=\min\left\{-P_{x}\right\}, \end{array}\right.\\ subject\;to\;\left\{\begin{array}{l} L_{j\min}\leq L_{j}\leq L_{j\max}\;\left(j=1,2\right),\\ P_{y}=L_{2}\cos\theta_{2}+d_{2}\cos\left(\theta_{2}+\alpha_{2}\right),\\ P_{y\min}\leq P_{y}\leq P_{y\max}. \end{array}\right. \end{array}$$

Based on (1) and (15), a series of points including (*P*_*x*min_, *P*_*y*_) and (*P*_*x*max_, *P*_*y*_) constitute the boundary of **W** during half a gait. In the same way, the feasible region of the waist during the following half a gait can be determined, which is symmetric with the previous feasible region about the central axis and has a translational distance in the climbing direction.

### 3.2. Effect of Structural Parameters

As mentioned above, the feasible region of the waist directly affects trajectory planning of the climbing robot. Both driving forces of active legs and the feasible region of the waist are related to the structural parameters of the robot, including body length ratio, driving angle, and waist spring coefficient. Therefore, the structural parameters should be reasonably selected, which contributes to a decrease in energy consumption and improvement in the stability of the climbing robot.

#### 3.2.1. The Effect of Body Length Ratio on Supporting Forces of Climbing Robot

To avoid the disturbance of unreasonable motion path to structural parameters, it is necessary to plan a smooth motion trail of the waist to highlight the effect of structural parameters on the climbing performance of the robot. In terms of control weaknesses of the single curve function, an acceleration function composed by piecewise continuous function is adopted, which combines advantages of trapezoidal function and sine function. In this case, the acceleration curve function transits smoothly. Thus, the inertia force of the system could be reduced, which effectively improves the climbing speed and stability.

Let *T* be the working time during half a gait. Here, let *L*_*Ij*_ and *L*_*Tj*_ (*j* = 1, 2) be the initial and terminal lengths of legs *L*_*j*_, respectively. According to kinematic analysis of the robot, *L*_*Ij*_ and *L*_*Tj*_ can be solved based on the start and terminal points (*x*_*P*_ and *x*_*PT*_) of waist trajectory at initial and terminal times.

To ensure the smooth path and no impact velocity of the GPL model, the following boundary conditions are listed. Namely, the corresponding velocity and acceleration of legs *L*_*j*_ are both equal to zero at initial and terminal times.

(16) $$\begin{array}{c} L_{j}\left(t_{0}\right)=L_{Ij},\\ {\dot{L}}_{j}\left(t_{0}\right)=0,\\ {\ddot{L}}_{j}\left(t_{0}\right)=0,\\ L_{j}\left(t_{m}\right)=L_{Tj},\\ {\dot{L}}_{j}\left(t_{m}\right)=0,\\ {\ddot{L}}_{j}\left(t_{m}\right)=0. \end{array}$$

Define the trajectory interpolation functions *s*(*τ*), and let *s*(*τ*) be the independent variable. Thus,

(17) $$\begin{array}{c} L_{j}\left(t\right)=L_{Ij}+\left(L_{Tj}-L_{Ij}\right)s\left(\tau \right),\\ {\dot{L}}_{j}\left(t\right)=\left(L_{Tj}-L_{Ij}\right)\dot{s}\frac{\tau }{T},\\ {\ddot{L}}_{j}\left(t\right)=\left(L_{Tj}-L_{Ij}\right)\ddot{s}\frac{\tau }{T^{2}}, \end{array}$$

where *s*(*τ*) meets the following constraints:

(18) $$\begin{array}{c} 0\leq s\left(\tau \right)\leq 1,\\ 0\leq \tau \leq 1,\\ \tau =\frac{t-t_{0}}{T},\\ T=t_{m}-t_{0}. \end{array}$$

Give priority to acceleration, and set $\ddot{s}\left(\tau \right)$ as follows:

(19) $$\begin{array}{c} \ddot{s}\left(\tau \right)=\left\{\begin{array}{ll} k\sin\left(4\pi \right)\tau , & 0\leq \tau \leq \frac{1}{8}\\ k, & \frac{1}{8}\leq \tau \leq \frac{3}{8},\\ k\sin\left(4\pi \tau -\pi \right), & \frac{3}{8}\leq \tau \leq \frac{5}{8},\\ -k, & \frac{5}{8}\leq \tau \leq \frac{7}{8},\\ k\sin\left(4\pi \tau -3\pi \right), & \frac{7}{8}\leq \tau \leq 1. \end{array}\right. \end{array}$$

Integrating (19) with respect to time and combining the initial and terminal conditions, the value of *k* can be determined as follows:

(20) $$\begin{array}{c} s\left(\tau \right)=\int_{t_{0}}^{t_{m}}\int_{0}^{1}\ddot{s}\left(\tau \right)d\tau ,\\ s\left(0\right)=0,\\ s\left(1\right)=1. \end{array}$$

According to (16), (17), (19), and (20) and structural parameters of the climbing robot (Table 1), the planned trajectory of the waist can be obtained. Combining the kinematic analysis of robot, the whole controlling function of legs can be confirmed.

**Figure 3:**
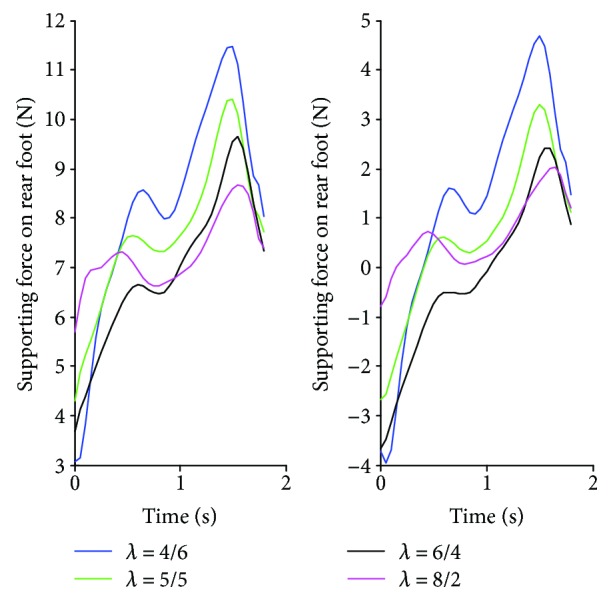
Supporting forces of the GPL model with different body length ratios.

To study the effect of the length ratio of front and rear body on driving forces of the robot, the different body length ratios *λ* = 8/2, *λ* = 6/4, *λ* = 5/5, and *λ* = 4/6 are set to calculate the driving forces according to the planned trajectory of waist and kinematic analysis. According to Figure 3, the supporting forces on front and rear feet present a significant decreasing trend as the increasing body length ratio. It is worth mentioning that the supporting forces on rear foot display a smaller negative value with the larger body length ratio, which contributes to a reduction in negative work of the robot. It suggests that the waist close to the bottom of the lower body can effectively optimize driving forces and system energy consumption.

#### 3.2.2. The Effect of Driving Angle on Start and Terminal Points of Waist Trajectory

According to bionic research [22, 23], the driving angle *β* of reptile gecko usually keeps a certain value. From (11), it can be shown that *β* has an obvious influence on the start and termination point space of the waist. To explore the effect of the driving angle on the start and terminal points of the waist, the driving angle range [8, 11] is selected to compute the start and termination point space and the feasible region of the waist based on the following dimension constraints and structural parameters (Table 1).

(21) $$\begin{array}{c} \left[\begin{array}{c} L_{1\min}\\ L_{2\min} \end{array}\right]\leq \left[\begin{array}{c} L_{1}\left(t\right)\\ L_{2}\left(t\right) \end{array}\right]\leq \left[\begin{array}{c} L_{1\max}\\ L_{2\max} \end{array}\right]. \end{array}$$

**Figure 4:**
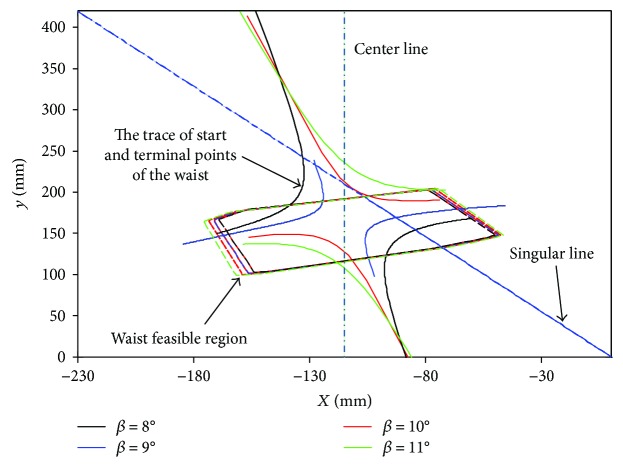
The start and termination point space and the feasible region of the waist.

From Figure 4, the range of the waist feasible region shows a slightly increasing trend with driving angle *β*. As mentioned above, trajectory planning should avoid the singular line due to waist joint on singular position, causing infinite force. Therefore, the start and termination points of the waist joint should be at the same side of the singular line. In addition, the motion trajectory must be within the waist feasible region in terms of structural constraint. Namely, the start and termination points of the waist also need to be located in the feasible region. Here, the optimal driving angle is 9°.

#### 3.2.3. The Effect of Waist Spring Coefficient on Supporting Forces of Climbing Robot

As we know, the reverse driving forces produce negative work, increasing system energy consumption. The driving forces are associated with the waist spring coefficient according to (3) and (5). Hence, the waist spring coefficient of the robot is analyzed to obtain a reasonable value, which contributes to a decrease in negative driving forces.

To optimize the energy consumption, we need to reduce thermal energy during robot climbing. It means that the negative work done by the supporting legs should be avoided. For a given waist trajectory or a climbing dynamic gait, the negative work will be as small as possible when the following (22) is satisfied. According to the equation, we can obtain the range of *k*_*w*_ (0.503–0.616) to ensure the positive driving forces.

(22) $$\begin{array}{c} F_{1}>0,\\ F_{2}>0. \end{array}$$

**Figure 5:**
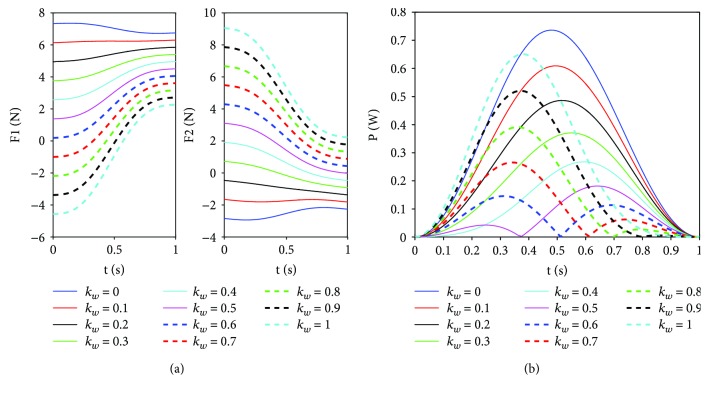
The effect of the waist spring coefficient. (a) The supporting forces on front and rear feet with different waist spring coefficients. (b) The system energy consumption with different waist spring coefficients.

Given the range of *k*_*w*_ (0–1 N · mm), the system energy consumption is calculated based on the above planning trajectory of the waist as well as (5) and (6).  Figure 5(a) shows that the supporting forces on front and rear feet decrease and increase with *k*_*w*_, respectively. There exist the largest negative and positive driving forces in the case of *k*_*w*_ = 1 N · mm than the ones in other cases. From Figure 5(b), it can be seen that the lesser negative forces active legs produce, the lesser the system energy consumption and the better the movement performance of the robot. Here, the reasonable spring coefficient can improve athletic performance while an oversize value of the spring coefficient may generate a negative effect on climbing motion of the robot. We can also find that the system energy consumption can obtain the smallest value when *k*_*w*_ = 0.5 N · mm. This is in accord with the above analysis based on (22). Hence, the value of *k*_*w*_ = 0.5 N · mm is selected in this paper.

### 3.3. Trajectory Optimization Based on Energy Consumption

During practical applications, there are high requirements of energy supply to be put forward for the robot system. Generally, the load-bearing capability of multilegged climbing robots is limited, which cannot carry greater weight energy storage units. Thus, it is significant to reasonably control mechanical energy consumption and improve climbing efficiency of the robot during the design process of the climbing robot. In this section, we focus on trajectory optimization based on system energy consumption.

Here, the waist joint coordinates can be expressed by the m-rank polynomial through the Hermite interpolation method, as follows:

(23) $$\begin{array}{c} x_{p}\left(t\right)=a_{j0}+a_{j1}\left(t-t_{0}\right)+\cdots +a_{jm}{\left(t-t_{0}\right)}^{m},\;j=1,\\ y_{p}\left(t\right)=a_{j0}+a_{j1}\left(t-t_{0}\right)+\cdots +a_{jm}{\left(t-t_{0}\right)}^{m},\;j=2. \end{array}$$

The above equation can be written in matrix form, as follows:

(24) $$\begin{array}{c} q\left(A,t\right)=\left[\begin{array}{c} x_{p}\left(t\right)\\ y_{p}\left(t\right) \end{array}\right]=Ah\left(t\right), \end{array}$$

where *A* is the multinomial coefficient matrix, which needs to be calculated.

(25) $$\begin{array}{c} A=\left[\begin{array}{ccc} a_{10} & \cdots & a_{1m}\\ a_{20} & \cdots & a_{2m} \end{array}\right]\in R^{2\times \left(m+1\right)},\\ h\left(t\right)={\left[\begin{array}{cccc} 1 & \left(t-t_{0}\right) & \cdots & {\left(t-t_{0}\right)}^{m} \end{array}\right]}^{T}. \end{array}$$

As in Section 3.2, the driving angle and body length ratio have been discussed and the optimal values have been obtained. The driving force curves of supporting feet at every start and termination points of the waist joint can be accomplished according to (1), (2), and (13). Considering the minimum driving force as the optimization goal, the best start point *q* (*A*, *t*0) and termination point *q* (*A*, *tm*) of the waist joint are selected. To ensure the smooth motion track of the robot, it is necessary to guarantee the angular velocity and angular acceleration to be continuous. Therefore, the following constraint equations need to be satisfied:

(26) $$\begin{array}{c} q\left(A,t_{k}\right)=\left[\begin{array}{c} x_{p}\left(t_{k}\right)\\ y_{p}\left(t_{k}\right) \end{array}\right]=\left[\begin{array}{c} x_{1k}\\ x_{2k} \end{array}\right]=Ah\left(t_{k}\right),\\ \dot{q}\left(A,t_{k}\right)=\left[\begin{array}{c} {\dot{x}}_{p}\left(t_{k}\right)\\ {\dot{y}}_{p}\left(t_{k}\right) \end{array}\right]=\left[\begin{array}{c} x_{1\left(k+1\right)}\\ x_{2\left(k+1\right)} \end{array}\right]=A\dot{h}\left(t_{k}\right),\\ \ddot{q}\left(A,t_{k}\right)=\left[\begin{array}{c} {\ddot{x}}_{p}\left(t_{k}\right)\\ {\ddot{y}}_{p}\left(t_{k}\right) \end{array}\right]=\left[\begin{array}{c} x_{1\left(k+2\right)}\\ x_{2\left(k+2\right)} \end{array}\right]=A\ddot{h}\left(t_{k}\right)\;\left(k=0,m\right), \end{array}$$

where *x*_*ij*_ is the boundary condition, which denotes the position, velocity, and acceleration of the waist at the start and terminal points.

Combining the above equation constraints, the boundary conditions can be obtained as follows:

(27) $$\begin{array}{c} {Ceq}_{j}\left(A\right)=0,\;j=1–6. \end{array}$$

In addition, the waist joint coordinates are limited to the dimension constraint relation as in (15). Thus, there are four inequality constraints as follows:

(28) $$\begin{array}{c} C_{i}\left(A\right)\leq 0,\;i=1–4. \end{array}$$

In terms of constraint condition for waist joint coordinate *x* or *y*, there are six equation constraints. And there are twelve equations and four inequalities for the waist joint coordinate in *x* and *y* directions. Therefore, at least seven undetermined coefficients are needed to solve the solution. Here, *m* = 6.

According to the above theoretical analysis, the system energy consumption is mainly caused by active joints. So, the power consumption of the robot at any time can be represented by multinomial coefficient matrices *A* and *h*(*t*).

(29) $$\begin{array}{c} P\left(A,t\right)=\overset{2}{\underset{j=1}{\sum }}P_{j}\left(t\right)=\hat{D}\ddot{q}\left(A,t\right)+\hat{H}\dot{q}\left(A,t\right)+\hat{S}q\left(A,t\right)+\hat{C}. \end{array}$$

The optimization problem of system energy consumption can be described as

$$\begin{array}{cc} \text{ (30)} & \underset{A}{\mathrm{Minimize}\to }\int_{t_{0}}^{t_{m}}P\left(A,t\right)dt,\\ subject\;to\;\left\{\begin{array}{c} {Ceq}_{j}\left(A\right)=0,\\ C_{i}\left(A\right)\leq 0. \end{array}\right. \end{array}$$

**Figure 6:**
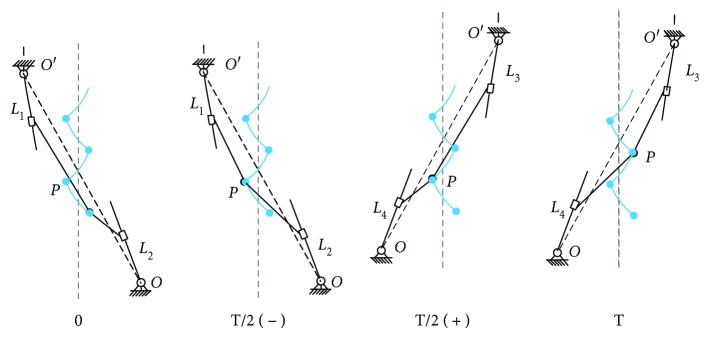
The optimal trajectory of waist during the whole gait. Here, the blue line and blue circle dot denote the optimal trajectory and the start and terminal points during the half gait, respectively.

So the trajectory optimization of the waist can be transformed into a nonlinear programming problem in the nonlinear equality and inequality constraints. According to the structural parameters and gait conditions, the multinomial coefficient matrices *A* can be solved and the optimal trajectory of the waist can be obtained, as shown in Figure 6.

## 4. Experiments and Results

### 4.1. Prototype Design Based on the GPL Model

**Figure 7:**
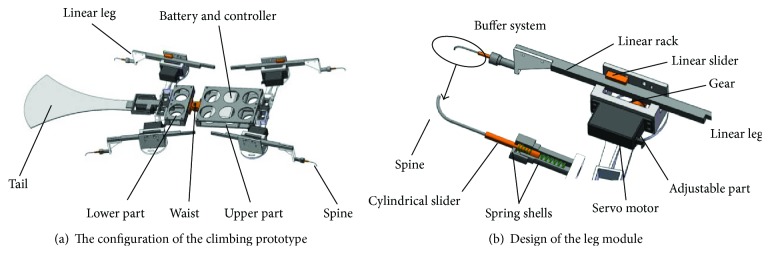
Prototype design based on the GPL model.

The robot based on the GPL model is composed of two rigid parts and four identical leg modules. According to Figure 7(a), there is no difference between mechanical configurations of the upper and lower parts in addition to the size parameter. And both parts connect to each other by the passive waist, which is a rotational joint in practice. Here, the position of the waist joint can be adjusted along the central axis of the upper part. The linear leg modules fixed on the rigid parts are adopted to realize the desired motion of the climbing robot, and they keep a certain angle with the body axis. In order to facilitate parameter adjustment, the angle and position of the rotating joint can be regulated manually. From Figure 7(b), the linear leg module consists of spines, buffer units, a linear guide with a rack, and a linear slider fixed on the rigid part. Here, the spine is fixed on the cylindrical slider that can slide in a linear track. The track named as spring shells is divided into two segmentations by a cylindrical slider, which plays a buffer role in two directions. To acquire the accurate motion of the robot, the linear leg modules are directly driven by a rotary motor with a rack and pinion mechanism. The corresponding linear speed of linear legs can be regulated and controlled by servo motors.

In view of the overturning torque in the whole climbing process, it is necessary to reduce the torque to avoid drop damage. As we know, the overturning torque becomes larger as the thickness of the robot increases. So, the thickness of the robot is limited to 14.8 mm and the motor is placed on the rigid parts. Moreover, a thin flat tail acting as a support is designed to obtain a more stable climbing movement. In fact, the tail is ignored in dynamics of the GPL model due to its micro weight. In addition, the controller and battery are located in the upper part, which have the effect of counter weight by adjusting their corresponding position.

### 4.2. Actual Climbing Experiments and Force Measurement on Supporting Feet

**Figure 8:**
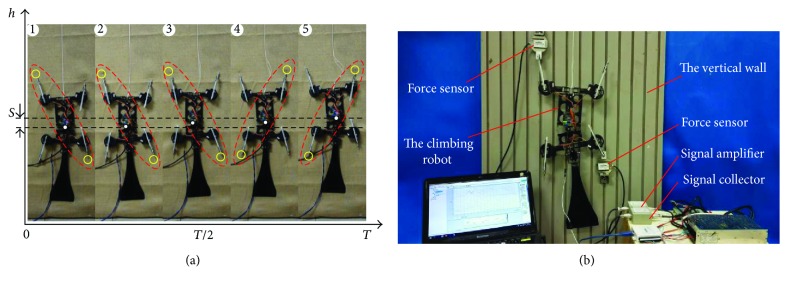
Actual climbing experiments on cloth-covered climbing surface and force measurement on supporting feet. (a) The sequence of climbing gait. Here, the red dotted line indicates the current adherent mechanism and the yellow circles indicate the adherent feet. (b) The test facilities of climbing robot and supporting force experiment.

In order to verify the designed gait and condition of gait reuse, the test platform with linen is established and the actual climbing experiment is conducted. According to Figure 8(a), the robot adopts a symmetrical gait and moves smoothly during a whole gait circle, which shows an intuitive understanding about gait reuse and continuity. It suggests that the theoretical analysis is validated and it provides the basis for the trajectory planning of the robot. It should be noted that the climbing speed is 66 mm/s in the dynamic analysis for robot climbing. Actually, the speed of the prototype is limited by failure of the spine's attachment/detachment to the cloth-covered climbing surface in the climbing experiment, which will be improved. Therefore, the speed of the prototype is reduced to 50 mm/s to maintain a more stable climbing in the test. Meanwhile, the force-measuring platform is built to explore the effect of structural parameters on supporting forces of the robot, as shown in Figure 8(b).

**Figure 9:**
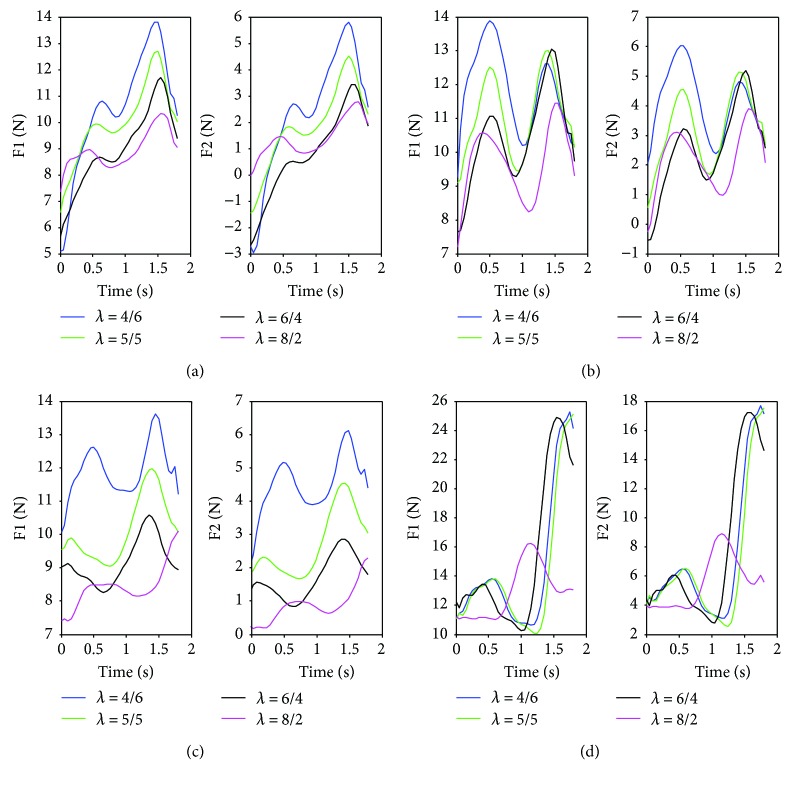
The supporting forces on front and rear feet with different waist spring coefficients and body length ratios: (a) *k*_*w*_ = 0 N · mm; (b) *k*_*w*_ = 0.2 N · mm; (c) *k*_*w*_ = 0.5 N · mm; (d) *k*_*w*_ = 1 N · mm.

In the supporting force measurement experiment, the planning trajectory of the waist in Section 3.2.1 is applied. And the ranges of the waist spring coefficient and body length ratio are in accord with the above analysis. From Figure 9, we can obviously find that the supporting forces on front and rear feet have the same decreasing trend with the increase in *λ*. Meanwhile, the ranges of supporting forces vary greatly with *λ* under a given waist spring coefficient *k*_*w*_. This indicates that the larger body length ratio contributes to a decrease in the driving forces, which is consistent with theoretical analysis. Based on (6), the system energy consumption is directly related to the supporting forces, which is expected to be as small as possible. Moreover, the waist spring coefficient has a significant effect on supporting forces, especially forces on rear feet. From Figures 9(a) and 9(b), there exist negative values on the rear-supporting foot when *k_w_* is 0 N·mm and 0.2 N·mm. According to Figures 9(c) and 9(d), the negative forces decrease gradually with the increase in *k*_*w*_ while the overall values of supporting forces become larger. Based on previous analysis, the negative forces on the rear-supporting foot should be avoided to reduce energy consumption in the process of climbing. In addition, there exist obvious fluctuations of supporting forces in Figures 9(a), 9(b), and 9(d). This means that the stability and structural strength of the climbing robot are easily damaged from the changing stresses to the actuators and manipulator structure. Therefore, 0.5 N·mm may be a more reasonable value of *k*_*w*_ in view of the whole positive amounts and smaller range of supporting forces on both feet.

### 4.3. Energy Consumption Experiments

**Figure 10:**
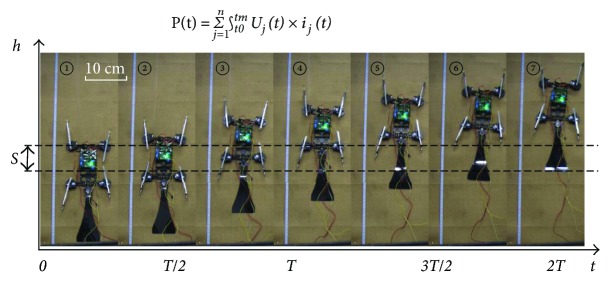
Energy consumption experiments of the climbing robot.

To verify the effectiveness of the optimized trajectory in Section 3.3, the energy consumption experiments are carried out, as shown in Figure 10. According to bionic research on gecko, the sinusoidal curve fitting the trajectory of the gecko's waist is applied to contrast with optimized trajectory [24, 26]. And the current sensors are adopted to obtain the instantaneous current values of motors. The instantaneous input power of the motor is the product of the instantaneous armature current and the instantaneous armature voltage. Thus, the total input power of the robot can be obtained as follows. Here, *U*_*j*_(*t*) is a constant 5 V.

(31) $$\begin{array}{c} P\left(t\right)=\overset{n}{\underset{j=1}{\sum }}\int_{t0}^{tm}U_{j}\left(t\right)\times i_{j}\left(t\right). \end{array}$$

**Figure 11:**
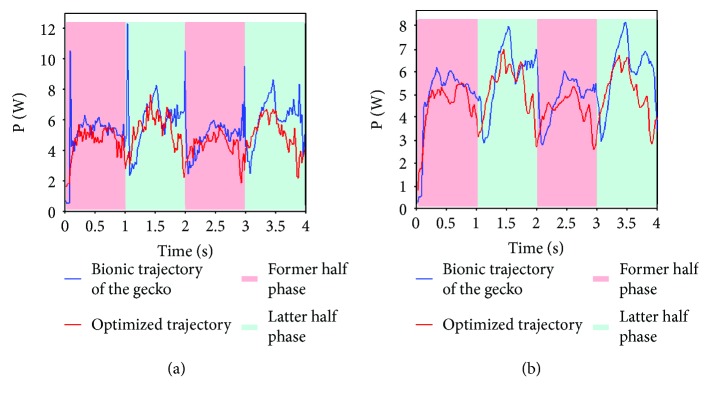
Energy consumption of the climbing robot with different trajectories. (a) The total input power of the robot with unfaltering data; (b) the total input power of robot with faltering data.

According to Figure 11, the power based on optimized trajectory is less than that based on bionic trajectory of the gecko. The former is 7.39 W and the latter is 9.79 W, in which the energy consumption based on optimized trajectory saves 24.5% than the latter one does. Obviously, it suggests that the optimized trajectory is effective, enhancing the theoretical analysis in this paper. Moreover, the results in our experiments may partly explain the reasonability of the quadruped reptile's kinesiology during dynamic gait.

## 5. Conclusion and Future Work

Benefitting from dynamic gait, the GPL model inspired by gecko has been proposed in our previous work. In order to further develop dynamic gait and reduce energy consumption of the climbing robot based on the GPL model, the gait design and trajectory planning are analyzed in this paper. In view of the special construction of the GPL model, the trot gait is adopted and the conditions of gait reuse are presented. According to the kinematics and dynamics of the GPL model, the structural parameters have a significant effect on the trajectory planning of the robot. The prototype experiment results show that the larger body length ratio and a certain elasticity of the waist joint contribute to a decrease in the supporting forces and reduction in system energy consumption, especially negative forces on supporting feet. Moreover, it suggests that the driving angle plays an important role on obtaining a reasonable performance of gait continuity. From the perspective of gecko, the pendular waist with certain elasticity is beneficial to storing kinetic energy in fluctuation and reducing energy consumption in the process of climbing. Therefore, the optimal trajectory of the waist is planned based on system energy consumption. The energy consumption experiments about optimal trajectory and gecko's sinusoidal curve are conducted, where a similar trend and less value of power suggest that the optimized trajectory is effective. Further, the results in our experiments partly explain the reasonability of the quadruped reptile's kinesiology during dynamic gait.

In the future work, the deformation of the structure will be considered in dynamics of the GPL model. And the flexible material and foot structure with multiple degrees of freedom will be included in the future design of climbing robots.

## Figures and Tables

**Table 1:** Structural parameters of the climbing robot.

Parameters	*L_a_* (mm)	*d*_1_ (mm)	*L*_1min_ (mm)	*L*_1max_ (mm)
Value	230	168	103	168
*m*_1_ (g)	*L_b_* (mm)	*d*_2_ (mm)	*L*_2min_ (mm)	*L*_2max_ (mm)
243	420	113	85	150
*m*_2_ (g)	*N_G_*	*η* (%)	*R_a_* (Ω)	*K_M_* (Nm/A)
223	2	77	4	10.2
